# Surveying immune and inflammatory alterations in periodontitis among individuals with Down syndrome: A preliminary cross‐sectional study

**DOI:** 10.1002/jper.70087

**Published:** 2026-02-19

**Authors:** Leticia Helena Theodoro, João Victor Soares Rodrigues, Marta A. A. Nuernberg, Pedro Henrique Petrilli, Mabelle de Freitas Monteiro, Valdir Gouveia Garcia, Rafael Scaf de Molon, Renato Correa Viana Casarin

**Affiliations:** ^1^ Center for Dental Assistance to Persons with Disabilities (CAOE) School of Dentistry Araçatuba SP Brazil; ^2^ Department of Diagnostic and Surgery School of Dentistry São Paulo State University (UNESP) Araçatuba SP Brazil; ^3^ CEUUN Francisco Beltrão Campus Unisep University Center Francisco Beltrão Paraná Brazil; ^4^ Department of Periodontics State University of Campinas (UNICAMP) Piracicaba SP Brazil; ^5^ Department of Implantology Latin American Institute of Dental Research and Education (ILAPEO) Curitiba PR Brazil; ^6^ Department of Periodontics and Endodontics School of Dental Medicine University at Buffalo Buffalo New York USA

**Keywords:** cytokines, Down syndrome, inflammation, innate immunity, interleukin, periodontitis

## Abstract

**Background:**

Individuals with Down syndrome (DS) are particularly vulnerable to early‐onset and rapidly progressing periodontitis, yet the local immune‐inflammatory alterations that contribute to this susceptibility are not fully understood. This study aimed to characterize cytokine profiles in gingival crevicular fluid (GCF) from individuals with DS and periodontitis and to compare them with those of periodontitis patients without DS and periodontally healthy controls.

**Methods:**

Forty‐five participants were enrolled and divided equally into three groups (*n* = 15 each): DS with periodontitis (DSP), periodontitis without DS (P), and systemically and periodontally healthy controls (C). GCF samples were collected from two interproximal sites per participant and analyzed for IL‐1β, IL‐4, IL‐6, IL‐10, TNF‐α, and IFN‐γ using multiplex immunoassays. Standard periodontal parameters, including probing depth, attachment level, bleeding on probing, and plaque index, were also recorded. Non‐parametric comparisons and multivariate approaches, including principal component analysis and hierarchical clustering, were employed to explore cytokine distribution patterns.

**Results:**

Individuals with DS and periodontitis exhibited a heterogeneous but predominantly pro‐inflammatory cytokine profile, characterized by elevated IL‐1β, IL‐6, TNF‐α, and higher Th1/Th2 and IL‐1β/IL‐10 ratios. In contrast, the P group demonstrated a Th2‐skewed response with higher levels of IL‐4, IL‐10, and IFN‐γ, suggestive of compensatory immune regulation. Healthy controls consistently displayed the lowest cytokine concentrations.

**Conclusion:**

This preliminary study identifies a distinct pro‐inflammatory cytokine profile in Down syndrome‐associated periodontitis, characterized by heightened IL‐1β responses and a Th1‐skewed immune pattern. While limited by sample size and the absence of a DS‐healthy control group, these findings provide initial mechanistic insights into the accelerated periodontal breakdown observed in DS and lay the groundwork for future, larger‐scale investigations.

**Plain language summary:**

People with Down syndrome (DS) often develop gum disease, called periodontitis, much earlier and more severely than others. This condition damages the tissues and bone that support the teeth and can lead to tooth loss. Researchers believe that an overactive immune system may play a role, but the exact reasons are not well understood. In this study, we examined the levels of several immune signaling molecules, called cytokines, in the fluid around the gums of people with DS and periodontitis, comparing them with individuals with periodontitis but without DS and with healthy participants. We found that people with DS and periodontitis had higher levels of inflammatory cytokines, showing an exaggerated immune reaction dominated by molecules that promote inflammation. In contrast, people with periodontitis but without DS showed signs of a more balanced immune response, including molecules that help to limit inflammation. These findings suggest that individuals with DS experience a stronger and less controlled inflammatory reaction in their gums, which may explain why their gum disease progresses so rapidly. Understanding these immune differences may help to develop better prevention and treatment strategies for people with Down syndrome.

## 1 INTRODUCTION

Periodontitis is a chronic multifactorial inflammatory disease initiated by a dysbiotic microbial biofilm and perpetuated by an exacerbated host immune response.^1^ The disease is characterized by destruction of periodontal ligament, cementum, and alveolar bone, and, if untreated, can lead to tooth loss.^2^ Although microbial dysbiosis is central to disease onset, it is now well recognized that individual variability in host immune‐inflammatory regulation is a decisive factor in disease severity and progression. Genetic, epigenetic, and systemic modifying conditions can alter immune responses, shifting the balance toward destructive inflammation.^3^

For pathogenic microbiota to colonize the periodontal sulcus or pockets, a dysbiosis of the resident microbiota must occur. Dysbiosis refers to an imbalance in the oral microbial community that disrupts its natural harmony and allows pathogenic species such as *Porphyromonas gingivalis*, *Tannerella forsythia*, and *Treponema denticola* to proliferate.^4^ This imbalance can be triggered by factors such as poor oral hygiene, systemic diseases, or impaired immunity. The resulting microbial shift promotes chronic periodontal inflammation and activates resident immune cells, including epithelial cells, dendritic cells, macrophages, and T‐helper subsets (Th1, Th2, Th17, Tregs).^5^ These cells act as sentinels, producing cytokines that regulate immune responses and drive the progression of tissue destruction.^6^

In some individuals, an exaggerated inflammatory response causes the body to overreact to minimal immune stimuli. This is common in people with immune‐mediated inflammatory diseases, such as psoriasis, rheumatoid arthritis, inflammatory bowel diseases, and Down syndrome (DS).^7^ Periodontal diseases are now recognized as multifactorial, influenced by genetics, environmental factors (e.g., smoking, alcohol, medications), and lifestyle.^4^, ^8^ Periodontitis is therefore a complex systemic disease, with progression and severity shaped by an individual's immune‐inflammatory profile.^1^, ^3^, ^9^ Consequently, its course varies among individuals, explaining the more aggressive progression in those with underlying immune or inflammatory abnormalities.^10^

Among systemic conditions, DS is particularly relevant to periodontal health. DS, caused by trisomy of chromosome 21,^11^ is associated with profound immune dysregulation, including chronic low‐grade systemic inflammation, impaired neutrophil chemotaxis, altered T‐ and B‐cell responses, and hyper‐reactivity of interferon signaling pathways encoded on chromosome 21.^11^, ^12^, ^13^ These abnormalities predispose individuals with DS to recurrent infections, autoimmune manifestations, and an exaggerated response to microbial challenges. Clinically, DS patients exhibit an early onset and accelerated progression of periodontitis, often disproportionate to their age and local plaque levels.^2^, ^14^ Consequently, periodontitis in DS is classified as a manifestation of systemic disease.^15^

Previous studies have examined systemic cytokine alterations in DS, with increased circulating levels of pro‐inflammatory mediators such as TNF‐α, IL‐1β, and IL‐6.^16^, ^17^, ^18^ However, systemic measurements may not accurately reflect the inflammatory milieu at the periodontal site, where local cytokine production directly mediates tissue destruction. Evidence regarding gingival crevicular fluid (GCF) cytokines in DS is sparse and fragmented, with only a few small‐scale investigations reporting inconsistent findings. Most prior studies have not compared DS patients with periodontitis with systemically healthy individuals with similarly severe forms of the disease, making it difficult to unravel DS‐specific immune alterations from those driven by periodontitis alone.

Therefore, there remains a significant knowledge gap regarding the local immunoinflammatory profile of DS‐associated periodontitis. Understanding whether DS patients exhibit distinct cytokine patterns at the site of periodontal breakdown is critical, as it may explain the disproportionately aggressive nature of periodontitis in this population. The present study aimed to analyze and compare GCF cytokine levels in individuals with DS and periodontitis, individuals with periodontitis but without DS, and healthy controls. By applying both univariate and multivariate approaches, this investigation sought to capture not only individual cytokine differences but also broader immune response patterns. In doing so, our study provides insights into how immune dysregulation in DS shapes periodontal inflammation.

## 2 METHODS

### 2.1 Study design

This study is a cross‐sectional observational clinical investigation comparing the immune‐inflammatory response patterns of individuals with DS and periodontitis with those without DS. The study was approved by the Research Ethics Committee in Human Research at the Faculty of Dentistry of Araçatuba (CAAE: 40589320.7.0000.5420) and by the Brazilian Register of Clinical Trials (REBEC) protocol #RBR‐5syrc3r. It was conducted at the Dental Assistance Center for People with Disabilities (CAOE) at the same University. All participants and their legal guardians were fully informed about the nature of the study and signed the informed consent form. For patients with DS, an assent form was also provided. The study was developed according to the STROBE guidelines for observational studies.

### 2.2 Patient recruitment and study group

Participants were recruited between March 2023 and March 2025 at the CAOE and the Periodontics Clinic of the School of Dentistry at Araçatuba, São Paulo State University (UNESP). Individuals with DS were referred to the study by clinicians at the CAOE during routine care and underwent periodontal examination to determine eligibility. Control participants without DS, including both periodontally healthy individuals and those diagnosed with stage III or IV grade C periodontitis, were consecutively selected from patients seeking treatment at the university's general periodontics clinic. All potential participants (or their legal guardians) were informed about the study objectives and procedures, and written informed consent (or assent for DS individuals) was obtained prior to enrollment. Eligibility was confirmed through a comprehensive medical and dental history, and periodontal status was determined via full‐mouth examination performed by one calibrated examiner (MAAN).

A total of 45 individuals were thus recruited and allocated into one of three groups based on the inclusion criteria.

Control Group (Group C; *n* = 15): Systemically and periodontally healthy individuals, characterized by: absence of clinical attachment level (CAL), probing pocket depth (PPD) < 3 mm, no radiographic bone loss, and aged 18 years or older.

Periodontitis Group (Group P; *n* = 15): Systemically healthy individuals with a diagnosis of stage III or IV grade C periodontitis, aged 18 years or older, and with a minimum of 20 teeth.

Down Syndrome Group (Group DSP; *n* = 15): Individuals with a diagnosis of DS and stage II or III grade C periodontitis, aged 18 years or older, and with a minimum of 20 teeth.

Exclusion criteria included: current or past smoking habits; pregnancy or lactation; use of antibiotic or anti‐inflammatory medications within 6 months prior to the study; prior periodontal treatment within 6 months before sample collection; less than 20 teeth, and diabetes or other systemic diseases affecting the immune‐inflammatory process.

It is important to mention that a group with DS and periodontal health were not included in our cohort. This group would provide normal baseline levels of cytokines allowing us to see the changes that occur in DS patients due to the presence of periodontitis. In our clinical setting, the vast majority of adolescents and adults with DS present with established gingival inflammation or periodontal breakdown, consistent with the very high prevalence reported in the literature. As a consequence, DS individuals who meet strict criteria for periodontal health (PPD ≤ 3 mm, no CAL, and no bleeding) are challenging to find at our center making their inclusion impractical for this study Therefore, our study should be viewed as a pilot investigation focused on understanding local cytokine patterns in DS individuals who already manifest the disease phenotype most representative of this population. Therefore, without data from periodontally healthy DS individuals a causal inference is not feasible based on the current dataset.

### 2.3 Clinical parameters

The following clinical parameters were evaluated: plaque index (PI‐present or absent), PPD, bleeding on probing (BOP), and CAL. These evaluations were performed at six sites of all teeth in the oral cavity using a calibrated periodontal probe (PCPUNC‐15, Hu‐Friedy, Chicago, IL, USA). All clinical examinations were conducted by a single calibrated examiner (MAAN) to diagnose individuals with or without periodontitis. Prior to the evaluations, calibration procedures were carried out by a blinded examiner. For calibration, 10 individuals were examined twice, with a 1‐week interval between examinations. PPD and CAL were analyzed at six sites per tooth, and the obtained data were submitted to the Kappa test (0.88), ensuring intra‐examiner agreement.^19^

### 2.4 Analysis of immune‐inflammatory markers

Gingival crevicular fluid (GCF) was collected from all participants at two interproximal sites with PPD > 4 mm and < 6 mm with BOP in individuals diagnosed with periodontitis. In individuals without periodontitis, fluid samples were collected from two interproximal sites with 3 mm PPD and no BOP. To ensure sample integrity and to minimize contamination, sites presenting profuse bleeding were excluded. All sampling sites were carefully isolated with sterilized cotton rolls and gently air‐dried prior to collection. The supragingival bacterial biofilm was meticulously removed, and sterile paper cones (Tanari, Manacapuru, AM, Brazil) were inserted into the gingival crevice for 30 s only after bleeding had subsided. These procedures, adapted from established GCF collection protocols,^20^, ^21^ effectively reduce the risk of blood contamination and provide reliable cytokine measurements. The paper cones were then placed into microcentrifuge tubes (Eppendorf), coded for each individual and periodontal pocket, and 150 µL of phosphate‐buffered saline with 0.05% Tween‐20 was added. The samples were stored at –80°C for later analysis, as described previously.^17^, ^18^

Before analysis, fluid samples were diluted in 60 µL of buffer from the Millipore kit, vortexed for 30 min, and centrifuged for 10 min at 10,000 rpm. Aliquots of each gingival fluid sample were analyzed for IL‐1β, IL‐4, IL‐6, IL‐10, TNF‐α, and INF‐γ using Luminex/MAGpix technology. The analyses were performed in 96‐well plates using high‐sensitivity panels (Millipore Corporation, Billerica, MA, USA), following the manufacturer's instructions. The concentration of each analyte was expressed in pg/mL. Samples with concentrations below the detection limit were recorded as “zero,” and those above the quantification limit were recorded with the highest value of the standard curve.^20^, ^21^ Immunomarker analyses were conducted by an examiner blinded to clinical examination and patient group allocation (RCVC). For a broader understanding of the immune response, cytokines were categorized based on their functional characteristics. The Th1 cytokine group, representing pro‐inflammatory cytokines associated with cellular immune responses, included IL‐1β, IL‐6, TNF‐α, and IFN‐γ. Conversely, the Th2 cytokine group comprised cytokines primarily involved in anti‐inflammatory and humoral immune responses, specifically IL‐4 and IL‐10. To quantify these responses, composite Th1 and Th2 scores were generated by summing the concentrations of their respective cytokines. The Th1/Th2 ratio was subsequently calculated to assess the balance between pro‐inflammatory and anti‐inflammatory immune responses.

### 2.5 Statistical analysis

All statistical analyses were performed using JMP Pro 13.0.0 (SAS Institute Inc.) with a significance level set at 5%. For each variable, the Shapiro‐Wilk test was applied to assess the normality of distribution. Given the small sample size per group (*n* = 15), non‐parametric tests were prioritized to ensure robustness. While a formal a priori power analysis was not feasible due to the scarcity of published quantitative cytokine data in DS populations, our study was designed as a pilot, hypothesis‐generating investigation consistent with prior exploratory immunological studies in rare populations. The use of non‐parametric testing, complemented by unsupervised multivariate approaches such as principal component analysis (PCA) and hierarchical clustering, was intended to maximize interpretability despite limited sample size. Demographic and clinical parameters (e.g., age, PPD, CAL, BOP, PI): Kruskal–Wallis test was used for between‐group comparisons. Pairwise group comparisons were performed using the Mann–Whitney U test with Bonferroni correction. Categorical variables (e.g., sex, PI) were analyzed using Fisher's exact test. Cytokine concentrations were log‐transformed and subjected to multivariate analysis. Hierarchical clustering was conducted using the Ward method, representing bidirectional clustering (samples and immunological markers). PCA and factor analysis were employed to identify patterns among cytokine expression and study groups. For individual cytokines, non‐parametric tests (Kruskal–Wallis and Mann–Whitney U with Bonferroni correction) were used for group comparisons.

## 3 RESULTS

### 3.1 Demographic and periodontal clinical data

The analysis of clinical and demographic characteristics revealed notable variations among the three groups (Table 1). Participants in the DSP group were significantly younger than both non‐DS groups and presented the highest proportion of male individuals (60%), contrasting with the predominantly female composition of the C (13%) and P (20%) groups. These demographic imbalances reflect the real‐world distribution of DS patients treated in specialized centers, where younger males are disproportionately represented. Although sex and age differences may influence inflammatory responses, the exclusion of smokers, individuals with systemic comorbidities, and those using anti‐inflammatory or immunomodulatory medications helped to reduce potential confounding.

**TABLE 1:** Demographic characteristics comparison and periodontal clinical variables of the sample studied.

	C	P	DSP
Age (years ± SD)	32.7 ± 4.9	36.7 ± 4.2	25.1 ± 6.5*^#^
Sex (%male)	13%	20%	60%*^#^
PI (mean% ± SD)	26.8 ± 13.6	33.0 ± 14.4	69.4 ± 31.6*^#^
BOP (mean% ± SD)	10.1 ± 8.9	35.8 ± 11.3*	44.0 ± 27.8*
Full mouth PPD (mm ± SD)	2.0 ± 0.3	2.7 ± 0.2*	2.6 ± 0.6*
% sites with PPD > 5 (mean% ± SD)	0.0 ± 0.0	24.4 ± 12.9*	7.5 ± 17.7^#^

*Note*: #Indicates statistical difference to P group. Statistical analysis performed using Kruskal–Wallis test and Mann–Whitney U test with Bonferroni correction for continuous variables; Fisher's exact test for categorical variables. *p* < 0.05 considered significant. *Indicates statistical difference to C group.

Abbreviations: BOP, bleeding on probing; PPD, probing pocket depth; PI, plaque index; SD, standard deviation.

Oral hygiene also varied substantially across groups. The DSP group exhibited the highest PI, with values more than double those of the non‐DS groups (69.4% vs. 26.8% and 33.0%; *p* < 0.05). BOP and PPD were significantly elevated in both periodontitis groups (P and DSP) compared with the healthy controls (C). As expected, the P group showed the highest percentage of sites with PPD > 5 mm (*p* < 0.05), reflecting more extensive periodontal breakdown.

### 3.2 Cytokine profile

Intergroup comparisons of gingival crevicular fluid biomarkers demonstrated distinct inflammatory profiles (Table 2). The P group exhibited higher concentrations of IFN‐γ (*p* = 0.04), IL‐10 (*p* = 0.00), IL‐4 (*p* = 0.05), and aggregated Th2 levels (*p* = 0.05) compared with the DSP group. In contrast, the DSP group showed higher Th1/Th2 and IL‐1β/IL‐10 ratios (*p* = 0.05), suggesting a relative shift toward a more pro‐inflammatory environment.

**TABLE 2:** Comparison of cytokine levels among groups.

Evaluated variables (pg/mL)	C	P	DSP
IFN‐γ	19.1 ± 5.9	26.9 ± 14.4^#&^	17.9 ± 8.2
IL‐10	38.5 ± 20.7*	36.9 ± 23.6^#^	15.9 ± 6.1
IL‐1β	14.6 ± 11.9*	160.5 ± 283.7	847.3 ± 1549.7
IL‐4	20.3 ± 10.3	26.4 ± 17.4^#^	16.4 ± 8.0
IL‐6	12.6 ± 7.0	18.3 ± 6.6	21.9 ± 17.2
TNF‐α	20.3 ± 6.6*	24.9 ± 7.1^&^	24.6 ± 12.0
Th1	66.6 ± 21.8*	230.7 ± 286.4^&^	911.7 ± 1566.3
Th2	58.9 ± 29.3*	62.4 ± 32.8^#^	32.3 ± 12.7
Th1/Th2	1.4 ± 0.4*	5.4 ± 9.0^#^	22.1 ± 29.3
IL‐1β/IL‐10	0.4 ± 0.6*	8.0 ± 15.7^#^	41.2 ± 61.7
INF‐γ/IL‐4	1.2 ± 0.5	1.1 ± 0.4	1.2 ± 0.4

*Note*: ^#^Statistically significant difference between P and DSP (*p* < 0.05).

^&^Statistically significant difference between C and P (*p* < 0.05).

*Statistically significant difference between C and DSP (*p* < 0.05).

Notably, the most striking and clinically relevant finding was the marked elevation of IL‐1β in the DSP group, which reached a mean level of 847.3 pg/mL, more than five‐fold higher than the P group (160.5 pg/mL) and nearly 60‐fold higher than the healthy group (14.6 pg/mL). This pronounced IL‐1β increase strongly influenced the aggregated Th1 values and the Th1/Th2 balance in the DSP group, indicating an amplified upstream inflammatory response. Although other cytokines showed more modest variations across groups, the IL‐1β elevation uniquely distinguished the DSP inflammatory profile.

As expected, the healthy control group consistently exhibited the lowest cytokine levels (*p* < 0.05), with the exception of the IL‐6 and IFN‐γ/IL‐4 ratio, which showed no significant difference among groups.

### 3.3 Multivariate analysis

**FIGURE 1:**
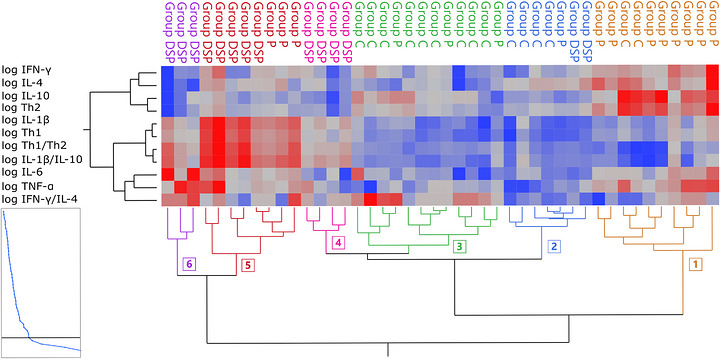
Hierarchical cluster analysis was used to cluster patients and subgingival cytokine patterns. The log of cytokine concentrations is represented in the heatmap, where red indicates higher concentrations of each cytokine in the patients and blue indicates lower concentrations of each cytokine. Six clusters of patients were identified and colored accordingly: orange (Cluster 1), blue (Cluster 2), green (Cluster 3), pink (Cluster 4), red (Cluster 5), and purple (Cluster 6). The patients were assigned to groups based on the conditions they presented: Group C (control), Group P (periodontitis), and Group DSP (Down syndrome and periodontitis). The distribution of the six distinct clusters in each Group is demonstrated in Figure 2, which reveals distinct immune patterns and a higher variability of inflammatory responses in patients with Down syndrome and periodontitis.

The multivariate analysis enabled the classification of the groups based on their cytokine profiles and identified that P and DSP presented different inflammatory patterns. The hierarchical cluster analysis classified the patients into six distinct clusters related to cytokine release, and those clusters efficiently divided the groups (Figure 1). Clusters 2 and 3 primarily described the C Group and were characterized by lower cytokine concentrations. The P group, however, was predominantly represented by cluster 1, characterized by high levels of IFN‐γ, IL‐4, TNF‐α, IL‐10, and Th2. The DSP group displayed high variability in inflammatory patterns, with a greater number of patients grouped in clusters 4, 5, and 6, where levels of IL‐1β, IL‐6, TNF‐α, Th1, Th1/Th2, and IL‐1β/IL‐10 were elevated.

**FIGURE 2:**
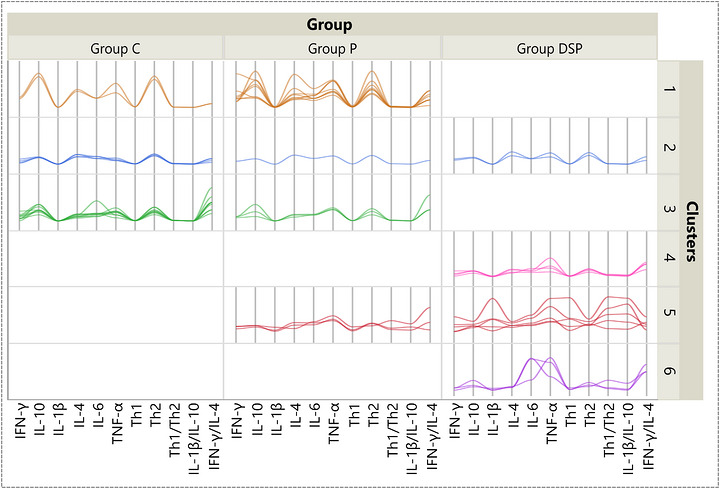
Cytokine expression profiles across different experimental groups and clusters. The six clusters were defined based on hierarchical cluster analysis (Figure 1). Each row corresponds to one cluster (Clusters 1–6), and the color of the lines indicates cluster membership: orange (Cluster 1), blue (Cluster 2), green (Cluster 3), pink (Cluster 4), red (Cluster 5), and purple (Cluster 6). Line plots depict the expression patterns of multiple cytokines across three groups: Group C (control), Group P (periodontitis), and Group DSP (Down syndrome + periodontitis). Individual lines within each panel represent separate samples. This visualization highlights differences in cytokine expression patterns between experimental groups and identifies clusters of co‐regulated cytokines.

The distribution of the six distinct clusters in each group is demonstrated in Figure 2, which reveals distinct immune patterns and a higher variability of inflammatory responses in patients with Down syndrome and periodontitis.

### 3.4 Principal component analysis

**FIGURE 3:**
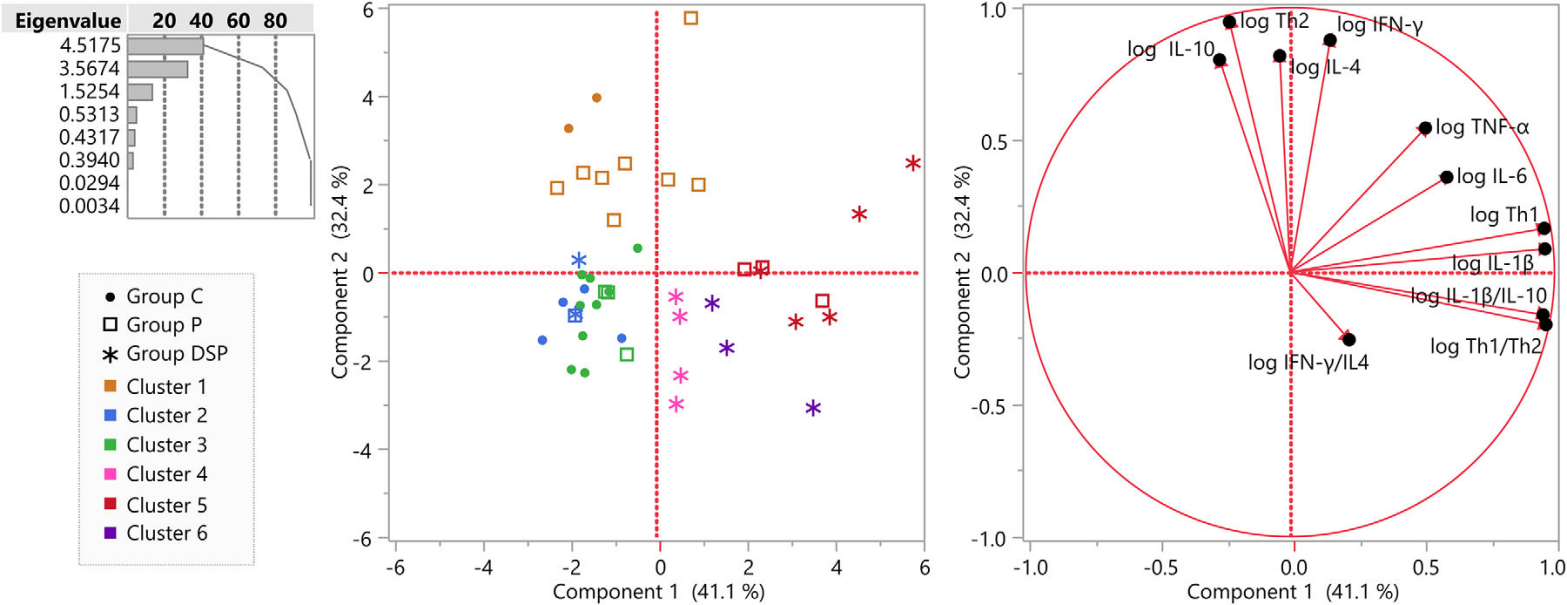
Principal component analysis (PCA) and factor analysis considering all cytokines and their ratios as variables. Each patient was represented by a symbol based on the group to which they belonged, and the colors of the symbols were defined based on hierarchical cluster analysis.

Additionally, PCA demonstrated the distribution of samples based on all analyzed variables (Figure 3). Components 1 and 2 accounted for 73.5% of the variability in the study variables, and, interestingly, they were able to discriminate the differences between groups (indicated by symbols on the graph) and clusters (represented by colors) based on the inflammatory response. Component 1 captured most of the differences between DSP and C, with DSP positively correlated with IL‐1β, TNF‐α, and IL‐6. Component 2 highlighted the distinctions of P to DSP and C, and P was positively correlated to IL‐10, IL‐4, and INF‐γ.

**FIGURE 4:**
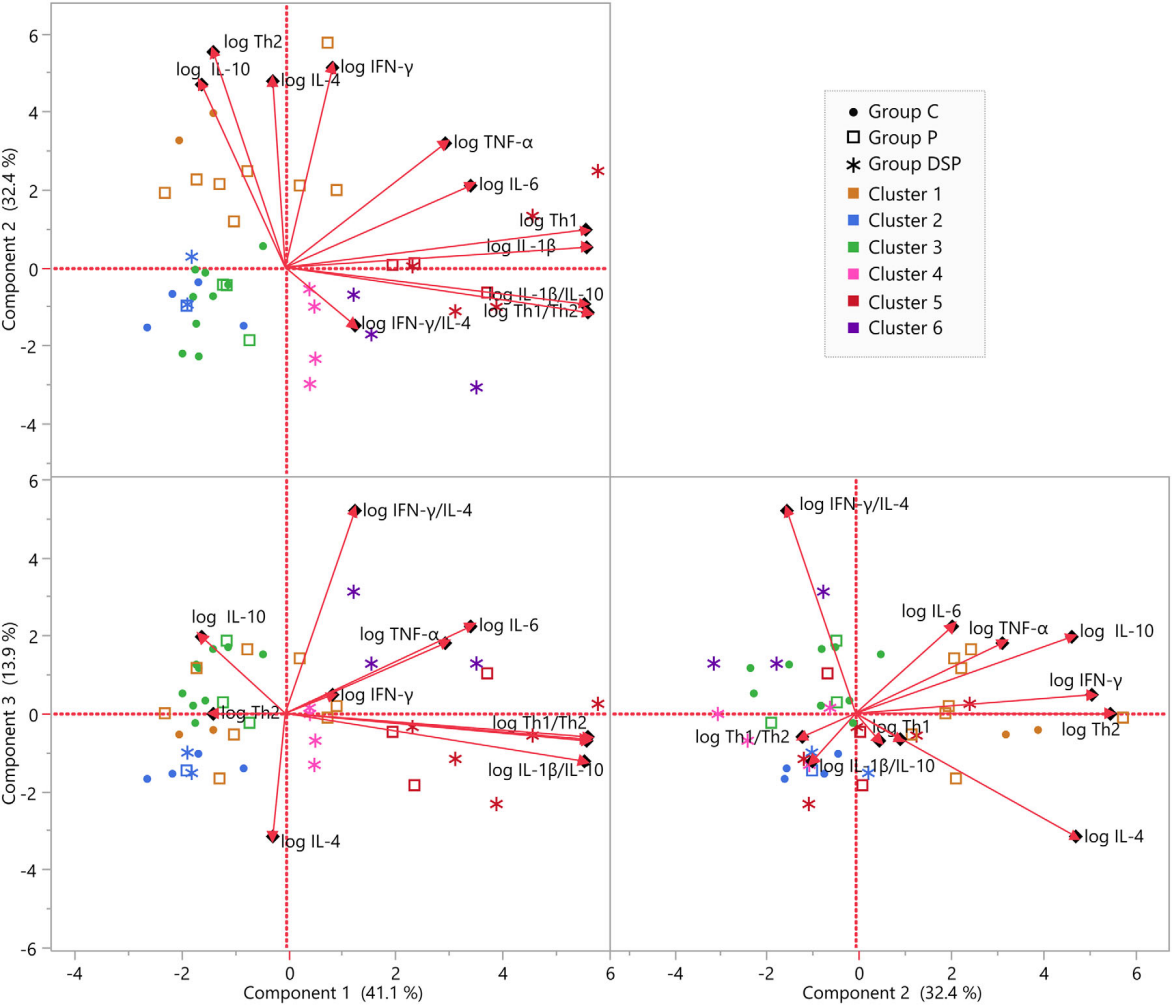
Principal component analysis (PCA) describing the components 1, 2, and 3.

Notably, some heterogeneity in the inflammatory response within the P group led to the grouping of some patients with either the C or DSP groups. The role of component 3 in differentiating the groups is described in Figure 4.

## 4 DISCUSSION

The results of the present study indicate that individuals with DS affected by periodontitis exhibit considerable variability and significant heterogeneity in their cytokine profiles within periodontal pockets, demonstrating a pronounced pro‐inflammatory pattern when compared with the other groups. This finding highlights the complex immune response in DS individuals, particularly in relation to periodontal disease. Although our findings indicate that individuals with DS and periodontitis may exhibit a tendency toward a Th1‐dominant cytokine profile, we emphasize that these observations are exploratory and should be interpreted with caution. The cross‐sectional nature of the study and the demographic and clinical imbalances, particularly the absence of a periodontally healthy DS group, differences in disease severity, and variations in sex distribution and oral hygiene, limit our ability to draw strong causal inferences. Rather than attributing the cytokine differences exclusively to DS‐related immune dysregulation, our results support a broader and more measured hypothesis: individuals with DS may mount an amplified, yet heterogeneous, local inflammatory response to periodontal disease. This hypothesis warrants confirmation in larger, demographically balanced cohorts that include DS‐healthy controls and allow for clearer delineation between baseline and disease‐related immune characteristics.

Down syndrome has long been associated with periodontitis, which tends to manifest early and to progress aggressively within this population.^22^ The systemic and immunological factors in individuals with DS, including altered immune responses, are thought to significantly influence the development and progression of periodontitis. These factors, however, do not fully account for the severity and rapid progression of the disease, as periodontitis is multifactorial in nature and influenced by various factors, many of which are altered in individuals with DS.^23^, ^24^ For instance, DS patients exhibit immune dysregulation, including chronic low‐grade inflammation and abnormal cytokine responses, which contribute to increased susceptibility to periodontal disease. While it is well established that individuals with DS have a chronic pro‐inflammatory state, characterized by aberrant cytokine levels, most previous studies have primarily focused on cytokines found in plasma, which may not fully reflect the local inflammatory environment in periodontal tissues.^11^ By investigating the inflammatory cytokine profile directly in GCF, collected from the gingival sulcus or periodontal pockets, this study provides a more localized and direct insight into the inflammatory processes occurring at the site of disease. This approach not only provides valuable insights about the unique immune response in DS but also enhances our understanding of how these local immune factors may drive the progression of periodontitis in this vulnerable population.

Our findings revealed higher concentrations of inflammatory cytokines in both the P and DSP groups compared with the healthy control group (C), indicating an elevated inflammatory state in individuals with periodontitis and DS. However, notable differences in the distribution of these cytokines were observed between the P and DSP groups. Specifically, individuals with grade C periodontitis (P group) exhibited higher levels of IFN‐γ, IL‐4, TNF‐α, IL‐10, and Th2 cytokines. The elevated levels of IL‐4 and Th2 cytokines in the P group suggest a distinct immune profile, characterized by a predominant Th2 response. Th2 cytokines, such as IL‐4, are typically associated with anti‐inflammatory processes and help to regulate the immune response to control overactive inflammation. The higher concentrations of IL‐4 in the P group may reflect an attempt by the immune system to counterbalance the pro‐inflammatory effects of Th1 cytokines, such as IFN‐γ and TNF‐α, which were also found to be elevated in this group. This Th2 skewed response could be part of a compensatory mechanism in individuals with periodontitis, wherein the immune system tries to limit the damage caused by the chronic inflammatory state. IL‐10, another cytokine found to be elevated in the P group, is a well‐known anti‐inflammatory cytokine that helps in maintaining immune homeostasis and preventing excessive tissue damage. The presence of these anti‐inflammatory cytokines in the P group suggests a complex immune regulation occurring in response to periodontitis, where both pro‐inflammatory and anti‐inflammatory cytokines are involved in the disease process. In contrast, the DSP group exhibited a markedly amplified inflammatory profile, most notably driven by the exceptionally elevated IL‐1β levels, a several‐fold increase compared with both non‐DS periodontitis patients and healthy controls. IL‐1β is a key upstream mediator of innate immunity, promoting inflammation through its effects on epithelial cells, fibroblasts, endothelial cells, and leukocytes. It also plays a pivotal role in shaping adaptive immunity by supporting B‐cell proliferation and maturation. Importantly, IL‐1β is a potent stimulator of osteoclast activity and bone resorption, positioning it as a central cytokine in the pathogenesis of periodontal bone destruction.^25^, ^26^

This pronounced rise in IL‐1β, together with higher levels of IL‐6, TNF‐α, Th1 cytokines, and the elevated Th1/Th2 and IL‐1β/IL‐10 ratios, indicates a distinctly pro‐inflammatory environment characterized by dominant Th1 activation. The disproportionate IL‐1β response in particular suggests an intensified upstream inflammatory drive in individuals with DS. Collectively, these differences between the P and DSP groups highlight a more pronounced and potentially chronic inflammatory state in DS, which may help to explain the accelerated and severe periodontal breakdown often observed clinically in this population. However, rather than interpreting these findings as definitive evidence of a fixed immune polarization, they are better understood as reflecting the heterogeneity and possible hyper‐responsiveness of the DS immune system. Future studies with larger DS cohorts and the inclusion of periodontally healthy DS controls will be necessary to clarify whether IL‐1β overexpression represents an intrinsic DS‐related immune feature or a disease‐driven amplification.

As expected, the healthy control group displayed the lowest cytokine concentrations, consistent with the absence of periodontal inflammation. Overall, these results underscore the complex interplay of immune pathways in periodontitis and DS, where both Th1 and Th2 responses are activated, though in different magnitudes, and reveal distinct immune signatures across the groups.

One of the most striking differences between the P and DSP groups was the markedly higher levels of Th1 cytokines in the DSP group, which were 3.95 times higher than in the P group. This substantial increase could be attributed to the chronic bacterial infection and continuous periodontal inflammation associated with disease progression in both groups. However, the pronounced disparity between the two groups suggests that individuals with DS experience a chronic hyper‐inflammatory state, characterized by an exaggerated Th1 response. This hyper‐inflammatory condition results in a continuous, disproportionate production of cytokines, which is not entirely explained by the infectious stimulus alone. This finding aligns with previous studies, such as the work of Tsilingaridis et al., who observed higher concentrations of all cytokines, including Th1, in the gingival crevicular fluid of individuals with DS compared with a control group.^18^ This chronic overproduction of Th1 cytokines in DS may reflect an underlying immune dysregulation that leads to an exaggerated inflammatory response, further exacerbating the progression of periodontitis.

Supporting these findings, a recent study by Mouchrek et al. demonstrated that individuals with DS and stage II–IV periodontitis exhibited more extensive periodontal involvement and higher levels of cytokines, including IFN‐γ, IL‐17a, IL‐1β, IL‐4, and IL‐6, compared with healthy controls.^17^ These findings collectively suggest that DS is associated with a distinct, heightened immune response to periodontal disease, characterized by an imbalance between pro‐inflammatory and anti‐inflammatory cytokines, which may contribute to the more severe and rapid progression of periodontitis in this population.

IFN‐γ, also known as type II interferon, plays a critical role in immune system modulation, being produced from the translation of the IFNAR2 gene located on chromosome 21.^11^ It activates macrophages and upregulates monocyte responses to bacterial lipopolysaccharides, leading to the production of pro‐inflammatory molecules such as prostaglandin E2 (PGE2), IL‐1β, and TNF‐α. Thus, it plays a crucial role in periodontitis progression and the destruction of periodontal tissue.^27^ Since IFN‐γ is produced from chromosome 21, its elevated concentration in the serum of individuals with DS may suggest an interferon pathway.^28^, ^29^ Interestingly, in our study, we observed less pronounced concentrations of IFN‐γ in the DSP group, with higher levels in the P group, although both groups exhibited high concentrations of TNF‐α. A similar result was found in a study analyzing mRNA samples from the gingival tissue of individuals with DS and periodontitis. The authors identified various gene expressions, including IFNAR2, but found lower expression levels in DS individuals compared with euploid individuals.^30^

What has particularly captured the attention of researchers is the relatively early onset and accelerated progression of periodontitis in individuals with DS.^31^ Chronic inflammation in DS is thought to drive an exaggerated immune response, impairing the body's ability to regulate tissue damage and to control microbial infections effectively, which accelerates the progression of periodontal disease. These findings highlight the concept that individuals with DS exhibit an altered immune system that is less effective in responding to periodontal pathogens, which may explain the increased susceptibility to severe periodontitis in this population.^17^ In light of this, we specifically selected young, systemically healthy individuals with grade C periodontitis, a stage characterized by severe and progressive loss of the periodontal apparatus, making it an ideal model for understanding the factors driving aggressive periodontal disease. Despite the systemic health of the selected individuals, who did not have other chronic conditions that might influence immune function, we still observed notable differences in the inflammatory profiles between this group and the others. These significant discrepancies further underscore the unique inflammatory response in individuals with DS, reinforcing the notion that the immune dysregulation seen in this population plays a key role in the early onset and accelerated progression of periodontitis, even in the absence of additional systemic health complications.

Interestingly, our study found high expression of IL‐10 in the P and C groups, while the DSP group did not exhibit the same expression for this cytokine, as shown in the cluster analysis. IL‐10 is an anti‐inflammatory cytokine that reduces inflammation by inhibiting pro‐inflammatory cytokines such as TNF‐α and IL‐6.^17^, ^32^ It is primarily expressed during acute inflammation, where the body's anti‐inflammatory mechanisms release large amounts of IL‐10 to prevent tissue damage, as seen in autoimmune diseases or chronic inflammatory conditions such as systemic lupus erythematosus and rheumatoid arthritis.^33^ The higher expression of IL‐10 in the P group can be seen as an attempt to suppress acute inflammation occurring in these individuals, particularly due to the manifestation and progression of aggressive periodontitis, now classified as stage III or IV periodontitis. However, this was not observed in the DSP group, possibly because individuals with DS have a generalized deficiency in their immune and inflammatory systems, lacking control over the inflammatory process. This leads to persistently elevated pro‐inflammatory cytokine levels and impaired lymphocyte and macrophage activity, which hamper their ability to fight infections.

Other studies report similar findings, where DS participants had lower anti‐inflammatory cytokine levels compared with the control group, even though both groups had periodontitis.^16^ The researchers concluded that individuals with DS have a negative anti‐inflammatory modulatory response, explaining the difficulty in controlling periodontitis in these individuals.^16^ On the other hand, a recent meta‐analysis including 19 studies with 957 patients with DS and 541 controls found no statistical differences in cytokine levels between the DS and control groups. Although no significant differences in circulating IL‐4, IL‐6, IL‐8, and IL‐10 levels were found between the DS and control groups, the authors acknowledged the limitation of small sample sizes in these studies comparing IL‐10.^34^

Contrary to our findings, Huggard et al. observed a significant increase in IL‐10 levels in the DS group (*n* = 114) compared with the control group (*n* = 60).^35^ It is crucial to consider several factors that might contribute to this discrepancy. First, the authors in Huggard's study analyzed cytokine levels in peripheral blood plasma, which may differ from the local cytokine expression observed in the periodontal tissues, as was the focus of our study. Additionally, the participants in the DS group of the Huggard et al. study were relatively young, with an average age of 5.7 ± 4.7 years, which is much younger than the average age of 25 years in our DSP group. The difference in age could play a significant role in the observed differences in IL‐10 levels, as immune system responses often vary between age groups, especially in individuals with DS. The immune system undergoes changes throughout life, and younger individuals may exhibit a more pronounced immune regulatory response compared with older individuals, which could explain the higher IL‐10 levels observed in their study. Furthermore, the presence of periodontitis and its severity can also influence cytokine profiles. Previous studies have shown that 85% of individuals with DS aged between 17 and 42 years had at least one site with PPD ≥ 4 mm,^36^ and 70.3% had one or more sites with PPD ≥ 4 mm,^37^ indicating that many individuals with DS in this age range have active periodontitis. This increased prevalence of periodontal pathology in our older DSP group could explain why our findings did not show the same elevated IL‐10 levels. Chronic periodontal inflammation and tissue destruction may alter immune responses, potentially leading to lower IL‐10 levels as the immune system shifts toward a more pro‐inflammatory state.

Another key pro‐inflammatory marker, IL‐6, was elevated in almost all analyses conducted in individuals with inflammatory diseases. IL‐6 is secreted by T cells and macrophages, and it acts as an important mediator in the immune system's response to infection and injury. IL‐6 not only stimulates the production of acute‐phase proteins during systemic inflammation but also contributes to the progression of chronic inflammation.^26^ In our study, IL‐6 expression was higher for most participants in the DSP group, differing from the other two groups. Elevated IL‐6 expression is noteworthy because it is one of the main markers for the acute phase of inflammation. Its production is directly proportional to TNF‐α production, and persistently elevated levels may lead to the development of autoimmune diseases and other inflammatory conditions.^38^ Similar findings were reported by Huggard et al.^35^ where cytokine levels in the plasma of individuals with DS were higher for nearly all cytokines, including IL‐6, compared with the control group. The authors also observed a potential correlation between elevated IL‐6 levels in a young population and the early onset of Alzheimer's disease, which affects individuals with DS, as this cytokine plays a crucial role in neurodegenerative conditions in the central nervous system.

The mechanistic link between DS and the altered inflammatory outcomes observed in this study can be partly explained by gene dosage effects resulting from trisomy 21. Chromosome 21 encodes multiple genes involved in immune regulation, including IFNAR1, IFNAR2, and IFNGR2, which are components of the type I and II interferon receptor signaling pathways. Overexpression of these genes leads to heightened sensitivity to inflammatory stimuli and interferon hyperactivity, which promotes sustained activation of immune cells such as macrophages and T lymphocytes.^11^, ^28^ This hyperactivation results in increased secretion of pro‐inflammatory cytokines, such as IL‐1β, TNF‐α, and IL‐6, and may explain the Th1‐polarized immune profile identified in individuals with DS and periodontitis. Additionally, impairments in regulatory pathways, such as reduced IL‐10 production and dysregulated neutrophil function, may hinder the resolution of inflammation and contribute to tissue damage. Collectively, these genetic and immunological alterations provide a plausible biological mechanism for the enhanced and chronic inflammatory response in individuals with DS.

This study has important caveats that should be considered when interpreting the findings. First, the relatively small cohort (*n* = 45) limits statistical power and reduces generalizability. Recruiting adults with DS and periodontitis is intrinsically challenging due to the low prevalence of DS, ethical considerations when involving a vulnerable population, and the clinical reality that few adults with DS are systemically healthy and periodontally unaffected. Consequently, the absence of a periodontally healthy DS group is a major limitation, as it prevents establishing baseline cytokine levels independent of disease. This constraint reflects the population treated in specialized centers, where adults with DS free of periodontal inflammation are challenging to find at our center. Nonetheless, our sample size is consistent with previous cytokine‐based DS studies, and we strengthened analytic rigor by using non‐parametric statistical tests supported by unsupervised multivariate methods (PCA and hierarchical clustering), which consistently discriminated between groups.

Second, demographic and clinical imbalances, particularly the higher proportion of males and younger age in the DSP group, as well as increased plaque accumulation, may have influenced cytokine expression. These differences mirror the characteristics of DS individuals seeking care and were not the result of recruitment bias. To mitigate potential confounding, cytokine ratios such as Th1/Th2 and IL‐1β/IL‐10 were analyzed alongside absolute values, although residual confounding from sex, age, and oral hygiene cannot be fully excluded.

Third, the markedly elevated and variable IL‐1β levels in the DSP group strongly influenced composite indices such as Th1 scores and Th1/Th2 ratios. While IL‐1β hyper‐responsiveness is biologically plausible given the interferon‐driven immune dysregulation described in DS, the heterogeneity observed suggests that inflammatory activation in DS is not uniform and may reflect multiple underlying pathways. These findings should therefore be interpreted cautiously.

Additionally, subgingival microbiological analysis and quantitative assessments of behavioral factors such as diet and oral hygiene were not included. Although microbial dysbiosis in DS has been well documented in previous studies^39^, ^40^ and our aim was to focus on host‐response mechanisms, integrating microbiological and lifestyle data in future investigations would help to clarify how local environmental factors interact with immunoinflammatory alterations.

Finally, differences in periodontitis staging between groups may raise concerns. The DSP group predominantly presented with stage II–III disease, whereas the non‐DS periodontitis group exhibited stage III–IV grade C profiles. This choice reflected clinically equivalent aggressive trajectories: individuals with DS typically manifest periodontitis earlier and progress rapidly even at lower stages, while stage III–IV grade C in systemically healthy adults reflects a comparably severe and rapidly progressing phenotype. Nonetheless, this mismatch may contribute to variability in the immunoinflammatory patterns observed.

Despite these limitations, the combined use of quantitative, ratio‐based, and multivariate analytical approaches enhances confidence in the biological relevance of the trends identified. Future studies involving larger, demographically matched cohorts and inclusion of DS‐healthy controls are essential to validate these findings and more clearly distinguish disease‐related from DS‐related immune signatures.

## 5 CONCLUSION

In conclusion, this study provides an initial exploratory characterization of local cytokine profiles in individuals with DS and periodontitis, compared with aggressive non‐DS periodontitis, using both conventional statistical analyses and multivariate immune profiling. The findings reveal a distinct pro‐inflammatory pattern in the DS‐associated periodontitis group, marked particularly by heightened IL‐1β responses and a shift toward Th1‐dominant activation, suggesting mechanisms that may contribute to the earlier onset and rapid progression of periodontal breakdown frequently observed in this population. However, these observations must be interpreted within the context of the study's limitations, including the small sample size, demographic imbalances, lack of a periodontally healthy DS comparator group, and the cross‐sectional design. These factors prevent firm causal conclusions and limit the ability to differentiate baseline DS‐specific immune characteristics from inflammation‐driven changes. Despite these constraints, this work addresses an important gap in the literature by offering novel insights into the host‐response signature of DS‐associated periodontitis. The exploratory immune patterns identified here provide a valuable foundation for future longitudinal studies with larger and demographically matched DS cohorts, including DS‐healthy controls, that are needed to validate these findings and to clarify the underlying immunopathological mechanisms.

## AUTHOR CONTRIBUTIONS

**Leticia Helena Theodoro**: Conceptualization and methodology; formal analysis and investigation; writing—original draft preparation; writing—review and editing; funding acquisition; resources; supervision. **João Victor Soares Rodrigues**: Conceptualization and methodology; formal analysis and investigation; writing—original draft preparation; writing—review and editing. **Marta A. A. Nuernberg**: Conceptualization and methodology; formal analysis and investigation; writing—review and editing. **Pedro Henrique Petrilli**: Conceptualization and methodology; formal analysis and investigation; writing—review and editing. **Mabelle de Freitas Monteiro**: Conceptualization and methodology; writing—review and editing. **Valdir Gouveia Garcia**: Conceptualization and methodology; writing—review and editing; resources. **Rafael Scaf de Molon**: Conceptualization and methodology; writing—original draft preparation; writing—review and editing; funding acquisition. **Renato Correa Viana Casarin**: Conceptualization and methodology; writing—review and editing; supervision. All authors reviewed the results and approved the final version of the manuscript.

## CONFLICT OF INTEREST STATEMENT

The authors declare that there are no conflicts of interest to disclosure related to this study.

## STUDY PROTOCOL

The study protocol (RBR‐5syrc3r) was registered in Brazilian Clinical Trials Registry (REBEC), available at https://ensaiosclinicos.gov.br/rg/RBR‐5syrc3r
